# A Targeted Approach to Post-Mastectomy Pain and Persistent Pain following Breast Cancer Treatment

**DOI:** 10.3390/cancers13205191

**Published:** 2021-10-16

**Authors:** Philip J. Chang, Arash Asher, Sean R. Smith

**Affiliations:** 1 Department of Physical Medicine and Rehabilitation, Samuel Oschin Comprehensive Cancer Institute, Cedars-Sinai Medical Center, Los Angeles, CA 90048, USA; arash.asher@cshs.org; 2 Department of Physical Medicine and Rehabilitation, University of Michigan, Ann Arbor, MI 48108, USA; srsz@med.umich.edu

**Keywords:** post-mastectomy pain, persistent pain in breast cancer, cancer rehabilitation, post-mastectomy pain syndrome

## Abstract

**Simple Summary:**

There are many causes of pain following treatment of breast cancer. Pain may be due to nerve damage, problems of the musculoskeletal system, or both. Frequently, multiple different problems may be present at the same time which can make it difficult to determine the exact cause(s) of pain. Identifying the anatomic pain generators is essential to direct appropriate treatment. The purpose of this review is to outline different sources of post-mastectomy pain and to provide recommendations for the treatment of each one.

**Abstract:**

Persistent pain following treatment for breast cancer is common and often imprecisely labeled as post-mastectomy pain syndrome (PMPS). PMPS is a disorder with multiple potential underlying causes including intercostobrachial nerve injury, intercostal neuromas, phantom breast pain, and pectoralis minor syndrome. Adding further complexity to the issue are various musculoskeletal pain syndromes including cervical radiculopathy, shoulder impingement syndrome, frozen shoulder, and myofascial pain that may occur concurrently and at times overlap with PMPS. These overlapping pain syndromes may be difficult to separate from one another, but precise diagnosis is essential, as treatment for each pain generator may be distinct. The purpose of this review is to clearly outline different pain sources based on anatomic location that commonly occur following treatment for breast cancer, and to provide tailored and evidence-based recommendations for the evaluation and treatment of each disorder.

## 1. Introduction

Breast cancer is the most common cancer in the world, accounting for 11.7% of all new cancer cases in 2020 [1]. Despite being the most prevalent, breast cancer only accounts for 6.9% of the world’s cancer related deaths largely in part due to innovations in screening, genetic sequencing, and targeted treatment modalities [1,2]. The improvements in therapeutics has led to improved survival rates of 91% and 84% at 5- and 10-years, respectively [3], with estimates of over 3.8 million survivors in the United States alone [4]. Most of these survivors are treated surgically with varying forms of mastectomy, breast-conserving surgery and lymph node dissection frequently resulting in the formation of chronic pain (defined as pain persistent or recurring for more than 3 months [5]) commonly labeled as post-mastectomy pain syndrome (PMPS) [6]. 

One of the first descriptions of PMPS was termed intercostobrachial nerve entrapment syndrome [7]. It was described as pain that would occur following mastectomy and involved the upper arm, shoulder and chest, was worsened by movement of the shoulder girdle and was characterized as dull, aching or burning with intermittent stabbing [7]. PMPS has grown to become a routine issue of breast cancer survivorship with significant implications on quality of life, physical function, and healthcare utilization. 

The vast majority of new breast cancer cases are diagnosed as local or regional with over 90% treated surgically [3] contributing to the development of chronic pain syndromes resulting in significant healthcare expenditures. One study in the United States calculated these costs as approaching $1 billion annually when accounting for office visits, medication use and work/productivity loss [8]. Not included within these costs is the physical pain and loss of function experienced by these patients. 

Following treatment for breast cancer, patients experience loss of upper limb range of motion (prevalence 2–51% [9]) [10,11,12], decreased strength (prevalence 17–33% [9]) [13,14], and decreased ability to perform activities of daily living [9,15,16]. Pain associated with these impairments reduces quality of life by negatively impacting physical autonomy, psychological well-being, and social relationships [17,18]. Given this, it is unsurprising that many patients receive chronic opioid therapy for pain control. Lee and colleagues tracked opioid use among cancer patients undergoing curative-intent surgery, with most patients undergoing surgery for breast cancer [19]. They found that the risk of new persistent opioid use among patients who were pre-operatively opioid naïve was 10.4% with an increase in risk to 15–21% in those undergoing adjuvant chemotherapy [19]. These risks continue amidst ongoing opioid crises in parts of Africa and the Middle East [20] and a situation in the United States which has only worsened since the COVID-19 pandemic [21,22,23,24].

The implications of chronic pain following treatments for breast cancer are vast. As persistent pain following breast cancer treatment encompasses a number of distinct disorders, we have written this narrative review to provide a practical approach to the evaluation and management of chronic pain from the perspective of the physiatrist. This manuscript identifies anatomic pain generators and describes the evidence for targeted interventions to reduce pain, improve quality of life, and potentially reduce the need for chronic opioid analgesia.

## 2. Terminology

The term post-mastectomy pain syndrome was coined in 1984 [25] and officially defined by the International Association for the Study of Pain in 1986 as “chronic pain commencing immediately or soon after mastectomy or removal of a lump, affecting the anterior thorax, axilla, and/or medial upper arm” [26]. Since then, there have been numerous other terms seeking to better characterize PMPS including post-axillary dissection pain [27], post-mastectomy neuropathic pain [28], post-mastectomy chronic pain [29], chronic pain after breast cancer treatment [30], persistent post-mastectomy pain [31], and post-breast surgery pain syndrome [32]. In 2016, Waltho and Rockwell proposed a novel definition of PMPS being pain that occurs after any breast surgery; is of at least moderate severity; possesses neuropathic qualities; is located in the ipsilateral breast/chest wall, axilla, and/or arm; lasts at least 6 months; occurs at least 50% of the time; and may be exacerbated by movements of the shoulder girdle [6].

This patchwork of terminology has likely contributed to a wide incidence of PMPS with reports ranging from 12–72% [9,18,33,34,35]. The larger incidence rates are likely capturing more than classic neuropathic etiologies of PMPS and as Tait et al. noted, the larger frequencies are likely including musculoskeletal causes of pain [36]. 

## 3. Etiologies of Chronic Pain after Breast Cancer Treatment

**Figure 1:**
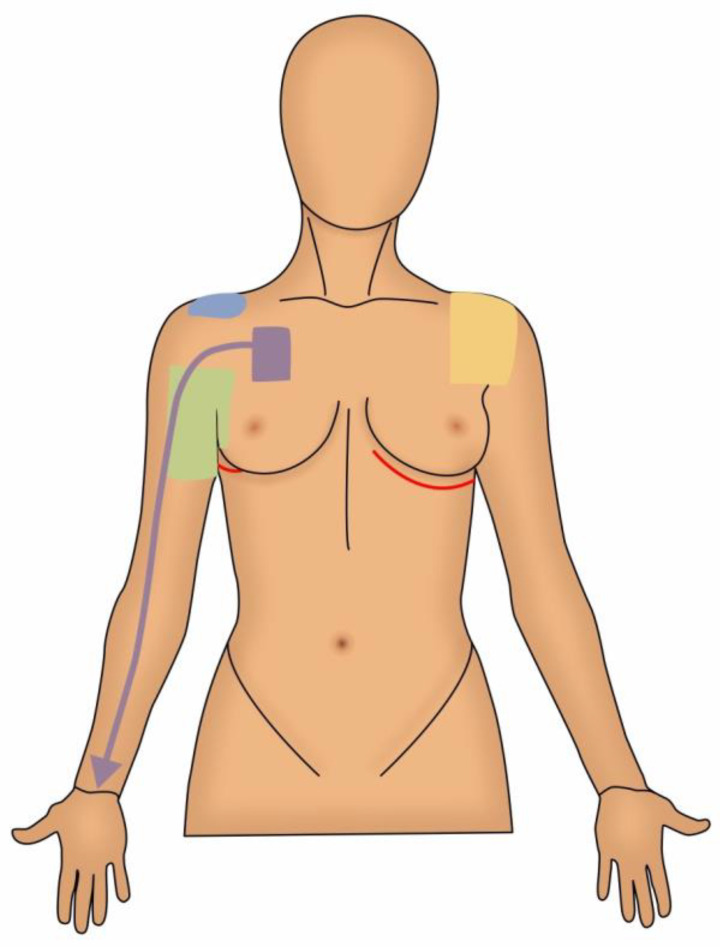
Pain Sources, Anterior View. Green: Intercostobrachial Nerve Injury; Blue: Shoulder Impingement Syndrome; Purple: Pectoralis Minor Syndrome/Neurogenic Thoracic Outlet Syndrome; Yellow: Adhesive Capsulitis; Red Lines: Intercostal and Intercostal Cutaneous Branch Neuromas Along Surgical Scars.

**Figure 2:**
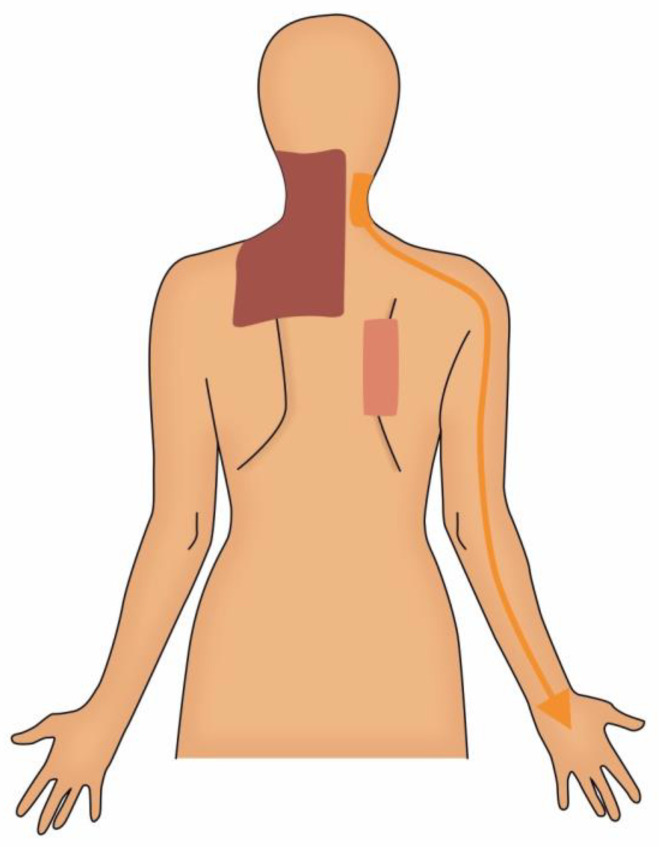
Pain Sources, Posterior View. Brown: Myofascial Pain; Orange: Cervical Radiculopathy; Pink: Scapulothoracic Bursitis.

The remainder of this review will focus on the common sources of pain following breast cancer treatment, categorized by neuropathic and somatic musculoskeletal sources (Figure 1 and Figure 2). For each potential source of pain we will review the underlying pathophysiology, diagnosis and treatment options. We will also briefly discuss nociplastic pain and its increasingly recognized role in breast cancer. Although there are numerous pain issues associated with breast reconstruction, they are beyond the scope of this review and will not be discussed here.

### 3.1. Neuropathic Sources of Pain

These may be considered as the classic etiologies of post-mastectomy pain, which occur due to nerve insult from surgery and/or radiation therapy. Recent reviews from Kokosis et al. and Chappell et al. have categorized etiologies of pain based off which nerves are injured building off the classification of neuropathic pain following breast surgery proposed by Jung and colleagues [28,32,37]. These nerves include most notably the intercostobrachial nerve, the anterior and lateral cutaneous branches of intercostal nerves T3–T6, the pectoralis medial and lateral nerves, the long thoracic nerve and the thoracodorsal nerve. Notably, the pectoralis, long thoracic and thoracodorsal nerves are all primarily motor nerves with injury resulting in loss of function to the pectoral, serratus anterior and latissimus dorsi muscles, respectively. As injury to these motor nerves does not result in typical neuropathic pain, they will not be discussed in this section, however, that weakness from motor nerve injury can lead to musculoskeletal pain manifesting as muscle spasms and dystonia that may be misinterpreted as neuropathic pain. Such musculoskeletal pain is sometimes distinguished in character as a squeezing or tightness and is further discussed in the myofascial pain section. For each pain source we will describe targeted interventional treatments as well as evidence-based treatment strategies. It should be noted that in many cases pain presents after surgery and continues to persist, however, in other situations pain may resolve and recur so chronologic onset of symptoms may vary. Furthermore, we acknowledge that various nerve blocks (including some of those listed in the following sections) are routinely used pre-emptively to prevent the development of chronic pain, however, these will not be discussed as the focus is on treatment of established chronic pain problems. Guidelines for treating general neuropathic pain utilizing oral and topical pharmacologic agents are shown in Table 1 and Table 2. 

#### 3.1.1. Intercostobrachial Nerve Injury

Pathophysiology: The intercostobrachial nerve (ICBN) usually arises from the lateral cutaneous branch of the 2nd intercostal nerve. It crosses the axilla into the upper arm where it innervates and provides sensation to the skin of the axilla and posteromedial upper arm [43]. Anatomic variations may cause symptoms to radiate distal to the elbow, including on the extensor forearm. Due to this positioning, the intercostobrachial nerve is extremely vulnerable to direct injury during axillary lymph node dissections with reported incidence ranging from 80–100% [44].

Diagnosis: Injury to the ICBN is diagnosed clinically. While numbness is very common, patients may have symptoms of neuropathic pain including sensations of burning, tingling, numbness, and electric sensations in the nerve’s distribution. On physical exam patients will have altered sensation and possibly a Tinel’s sign in the proximal, medial portion of the upper arm. 

Treatment: There is preliminary evidence for the use of nerve blocks in ICBN mediated pain. The ICBN can be readily identified and easily targeted with ultrasound given its superficial location. A small case series demonstrated improvements in pain ranging from 33–100% with relief lasting at least 4 weeks to 6 months [45]. Another case series noted a significant decrease in summed pain intensity scores, however, follow up was limited to one week [46]. More recently, a retrospective analysis demonstrated significant improvement in pain scores with various peripheral nerve blocks for post-mastectomy pain including ICBN blocks with durability of relief extending longer than 2 months in most cases. Notably, this review suggested that superficial serratus plane blocks at the 2nd or 3rd ribs could also be substituted in place of an ICBN block [47].

#### 3.1.2. Intercostal and Intercostal Cutaneous Branch Neuromas 

Pathophysiology: Neuromas form when peripheral nerves sustain injury during surgery and do not heal properly. The two main types of neuroma include terminal neuromas which occur when a nerve is completely transected, or a neuroma-in-continuity in which the nerve is still intact [48]. Intercostal nerves T3–T6 as well as their anterior and lateral cutaneous branches, which innervate the skin of the chest wall, are susceptible to direct injury and neuroma formation [28,32,49]. 

Diagnosis: Neuromas are typically identified clinically with symptoms of neuropathic pain along the affected nerve [50]. On exam, patients will have focal tenderness and may exhibit a Tinel’s sign with radiating symptoms along the distribution of the nerve. Sites of pain will frequently be along or in close proximity to surgical scar lines. Imaging can also play a role with evidence that ultrasound may be the best, albeit limited, modality for evaluation, as magnetic resonance imaging (MRI) and mammography have previously been shown to miss lesions [51]. During ultrasound examination, palpation with the transducer over the neuroma may reproduce symptoms and has been referred to as the “ultrasound trigger sign” [52]. Further confirmation can be achieved with a nerve block which often confers not only diagnostic but also potentially therapeutic benefit. 

Treatment: There is limited evidence that nerve blocks for neuroma-mediated pain can be an effective and potentially durable intervention. Tang and colleagues found that this simple intervention, in which they identified maximal areas of point tenderness and injected with a 2 mL mixture of 0.5% bupivacaine and 4 mg/mL dexamethasone followed by 1–2 min of massage gave significant benefits [53]. They injected 29 sites among 19 patients and found decrease in pain scores from 8–9/10 to 0–1/10 and most patients experienced long-term relief [53,54]. Another recent single-arm study using the same intervention identified 91 of these areas that the authors termed trigger points in 52 patients [50]. They found that a single injection achieved long-lasting relief of greater than 3 months in 72.3% of trigger points [55]. Pectoral nerve blocks, subcostal plane blocks, deep serratus plane blocks, erector spinae plane blocks, paravertebral nerve blocks and intercostal nerve blocks may also achieve similar effects and may be indicated when pain presents in a larger somatic distribution [47]. In cases of relief but recurring pain, surgical interventions may be considered. Surgical excision of neuromas and autologous fat grafting have been shown to be helpful in small trials [49,56,57]. Targeted muscle reinnervation, regenerative peripheral nerve interface and dermatosensory peripheral nerve interface surgeries in which damaged nerve endings are provided with a physiologic target allowing for axonal regeneration have also shown promise with studies in progress [58,59,60,61].

#### 3.1.3. Phantom Breast Pain

Pathophysiology: Phantom pain most commonly occurs in the setting of limb amputation but has also been reported following mastectomy. Phantom pain is thought to occur due to changes in the central nervous system, although peripheral and psychological factors may also play a role [62]. Phantom pain is distinguished from phantom sensations which constitute the non-painful continued experience of the presence of the breast following surgery and is reportedly more common [63]. Studies have reported incidence rates of 11.8–13.6% at one year although other studies report ranges as high as 40–50% [64,65].

Diagnosis: There are limited studies on phantom breast pain and strict diagnostic criteria are unavailable. However, other sources of pain should be ruled out and pain should appear to be emanating from a space no longer occupied by tissue. A previous study has stated the importance of distinguishing phantom pain from other causes of pain such as pain from the scar site [63]. A case study reported they were able to diagnosis phantom breast pain due to pain being felt in the absent breast with a lack of pain in the ipsilateral chest wall and arm [66].

Treatment: There have been no clinical trials on the treatment of phantom breast pain. Proposed treatment strategies have included the use of neuropathic pain medications (Table 1) and mirror therapy [66,67].

#### 3.1.4. Pectoralis Minor Syndrome/Neurogenic Thoracic Outlet Syndrome

Pathophysiology: Pectoralis minor syndrome is often classified as a subtype of thoracic outlet syndrome in which vasculature and/or accompanying neurologic structures are compressed either above or below the clavicle. In pectoralis minor syndrome, there is compression of the brachial plexus under the pectoralis minor muscle in the subpectoral tunnel [68]. It may account for a significant number of cases of thoracic outlet syndrome with one study reporting a prevalence of 22.6% among patients referred for treatment of thoracic outlet syndrome and was found to be the only cause of nerve compression in 6.1% of cases [69]. Although we are aware of no studies linking pectoralis minor syndrome and breast cancer, the authors attest to seeing it in clinical practice. Furthermore it has been demonstrated that decreased pectoralis minor muscle length is common following breast cancer treatment [70,71]. This muscle shortening compounded by further shortening and sclerosis that may occur in patients who undergo radiation therapy may create a predisposition to compression of the brachial plexus. Further study on the association between treatment for breast cancer and pectoralis minor syndrome is indicated.

Diagnosis: Pectoralis minor syndrome is most often diagnosed clinically. Patients will experience symptoms of pain, paresthesia, and weakness in the distribution of the pectoralis minor, shoulder, upper arm, forearm and hand. Although symptoms may occur in the distribution of nerve roots C5–T1, symptoms most commonly occur in the C8–T1 distributions and weakness will most often be present at the level of the hand [68]. Physical exam may be notable for sensory deficits in dermatomal distributions, hand weakness and pain with provocation. Provocative maneuvers include the elevated arm stress test, neck rotation, head tilt, Adson maneuver, and the upper limb tension test which has been reported to be the most accurate [68]. Electrodiagnostic testing can be performed however is not frequently obtained due to typically negative results and low sensitivity although abnormal measurements of the medial antebrachial cutaneous nerve may be found [72,73]. A more commonly used test to confirm diagnosis is a trigger point injection with lidocaine into the pectoralis minor, or chemodenervation with botulinum toxin which may also offer therapeutic benefit [73].

Treatment: The initial management is conservative with stretching of the pectoralis muscles and strengthening of the scapular retractor muscles. A unilateral self-performed stretch has been shown to be superior to assisted stretches and is easiest done against a wall or doorway [74]. Patients refractory to a self-directed program may trial a course of physical therapy for a supervised stretching/strengthening program and elastic therapeutic taping which may facilitate greater lengthening compared to stretching alone [75]. In addition to diagnostic value, trigger point injections and chemodenervation may provide significant relief and refractory cases may be referred to surgery for pectoralis minor tenotomy with or without brachial plexus decompression [73,76].

#### 3.1.5. Cervical Radiculopathy

Pathophysiology: Cervical radiculopathy is a condition in which the cervical nerve roots are affected most commonly through compression or less commonly through nondegenerative forces such as infection, infarction, avulsion, or tumor infiltration [77]. The incidence has been reported to be 107.3 per 100,000 in men and 63.5 per 100,000 in women peaking in the 4th and 5th decades of life [78,79]. C6 and C7 have been reported to be the most commonly affected roots with spondylosis causing 70% of cases [80]. While a specific link between cervical radiculopathy and breast cancer or treatment for breast cancer has not been demonstrated it is common and may be encountered in breast cancer patients [81].

Diagnosis: Cervical radiculopathy is first identified clinically and then further confirmed utilizing either imaging or electrodiagnostic testing. Patients typically present with unilateral neck pain radiating down the shoulder or arm with sensory or motor deficits in dermatomal distributions [80]. A thorough neurologic exam testing strength, sensation and reflexes should identify affected nerve roots. There are numerous provocative maneuvers that can be performed with the highest specificities for Spurling’s test, axial traction, Valsalva maneuver and the arm squeeze test [82,83]. One review reported a combination of a positive Spurling’s maneuver, axial traction and arm squeeze test increase the likelihood of cervical radiculopathy [82]. MRI is frequently used to confirm diagnosis and often imaging is used in conjunction with electrodiagnostic testing as complementary modalities [84,85]. Electromyography in particular has been shown to have good sensitivity and excellent specificity ranging from 87–100% [86].

Treatment: Conservative management is the mainstay of treatment and is often employed prior to obtaining imaging or electrodiagnostic testing in patients without red flags [87]. Conservative treatment may include physical therapy, modalities, non-steroidal anti-inflammatories, and muscle relaxants often for a period of at least six weeks [87,88]. Both manual and mechanical traction have evidence in reducing pain and to a lesser extent improving function and can be incorporated into a physical therapy program [89]. Cervical epidural steroid injections are a commonly used intervention and have been shown to be effective in providing short to intermediate-term relief and can help facilitate an exercise program [90,91,92]. Numerous surgical interventions are available with no evidence of superiority of a single procedure over another and limited evidence showing no long-term difference in pain outcomes with physical therapy [93,94].

### 3.2. Musculoskeletal Sources of Pain

There are numerous causes of musculoskeletal pain in the region of the upper limb, shoulder, and chest [81]. While the following sources of pain do not represent a comprehensive list, they do encompass the most frequently encountered problems that either exist commonly among the general population or occur with a greater frequency in the breast cancer population (Figure 1 and Figure 2). As these problems cause pain in similar distributions as classic neuropathic post-mastectomy pain, they may frequently be confused for or occurring concurrently with neuropathic PMPS. Therefore, it is essential to be able to differentiate between these different diagnoses as they have unique treatment strategies.

#### 3.2.1. Scapulothoracic Bursitis

Pathophysiology: Scapulothoracic bursitis is considered to be an underdiagnosed source of shoulder pain. It is most often associated as a potential sequelae of snapping scapula syndrome in which posterior shoulder pain, dysfunction and crepitus are present altering the motions of the scapulothoracic articulation [95,96]. Scapulothoracic bursitis is thought to occur secondary to overuse with chronic inflammation of the bursae in the superior or inferior medial borders of the scapula (supraserratus and infraserratus bursae), leading to fibrosis [97]. These disorders may result due to predisposing abnormal anatomy [97]. The altered biomechanics contributing to a protracted and depressed shoulder in breast cancer patients following treatment may increase the incidence of scapulothoracic bursitis. It has also been demonstrated that scapulothoracic bursitis is an underrecognized source of breast and chest wall pain [98]. Boneti et al. postulated that due to the proximity of the scapulothoracic bursa to intercostal nerves T2–6, breast and chest wall pain may be referred from scapulothoracic bursitis. They identified scapulothoracic bursitis as a significant source of breast/chest pain in 103 of 461 patients presenting with breast/chest wall pain. Of significance, 46.4% of those 103 patients had undergone partial or full mastectomy [98].

Diagnosis: Scapulothoracic bursitis and the snapping scapula syndrome are most often diagnosed clinically with the most common signs including medial scapular border tenderness, palpable crepitus and audible snapping [99]. On exam, the most common areas of tenderness will typically be at the superomedial border or inferior pole of the scapula and crepitus may be reproduced with shoulder abduction [100]. Pain may also be reproduced when applying an anterior directed force on the medial border of the scapula. Imaging is not routinely obtained although three-dimensional computerized tomography (CT) scans may detect bony irregularities and MRI may identify an inflamed bursa [97]. Corticosteroid injections with a local anesthetic are frequently used to confirm diagnosis [97,98].

Treatment: First-line treatment is typically conservative, consisting of rest, NSAIDs, activity modification and shoulder rehabilitation [101]. An exercise regimen should include strengthening of periscapular muscles, particularly the subscapularis and serratus anterior [101] and stretching of the pectoralis muscles. Patients refractory to conservative treatment may undergo injections into the scapulothoracic bursa. A retrospective review demonstrated that bursitis injections using a local anesthetic and corticosteroid provided complete pain relief in 83.5% of patients and partial relief in 12.6% of patients with only 3.9% of patients having no response [98]. A prospective open-label trial demonstrated that injection with steroid and hyaluronate once a week for three weeks significantly improved pain scores at all follow-ups from 1 week to 3 months [102]. Ultrasound guidance may not be strictly needed for such injections as a randomized controlled trial demonstrated no difference in pain scores from 1 week to 3 months between injections into the subscapularis and the scapulothoracic bursa although use of guidance to decrease the risk of pneumothorax may be considered [103]. In refractory situations, case studies have demonstrated benefit with surgical excision [104,105].

#### 3.2.2. Shoulder Impingement Syndrome

Pathophysiology: Shoulder, or subacromial, impingement syndrome is considered the most common cause of shoulder pain and contributes to multiple shoulder disorders [106,107]. Subacromial structures that may be affected include the biceps tendon, the subacromial bursa, and rotator cuff, although there is some controversy as to whether rotator cuff pathology occurs secondary to extrinsic compression vs. intrinsic degeneration [108]. In breast cancer patients, tightening of the pectoral muscles is common and can occur through a combination of mechanisms including pain induced contraction and radiation fibrosis [109]. This misalignment has been demonstrated and likely creates a predisposition to impingement as one study showed positive impingement signs in 13/25 breast cancer survivors vs. 0/25 controls [110,111].

Diagnosis: Shoulder impingement syndrome is primarily a clinical diagnosis. Presenting symptoms include pain in the lateral and anterior shoulder worsened with overhead movement [112]. Numerous provocative maneuvers can be performed and research suggests that multiple positive maneuvers results in greater accuracy [113,114]. One review recommends a combination of the Hawkins-Kennedy test, the painful arc test and the infraspinatus muscle strength test [113]. Imaging cannot confirm the diagnosis however it may be useful in assessing for tears and other shoulder pathology with the preferred modalities being ultrasound and MRI [115].

Treatment: Shoulder impingement syndrome is widely treated non-operatively although some authors recommend early referral to orthopedics if there is suspicion for a significant rotator cuff tear, labral tear or for high performance athletes [106]. NSAIDs and icing are often used for pain control and patients may be referred for physical therapy [112,113,116]. Physical therapy will often incorporate multiple modalities including heating/icing, manual therapy, and specific exercises focusing on strengthening of the rotator cuff and scapular stabilizers which has been shown to improve pain and disability [113,117]. Patients with continued symptoms after 4–6 weeks may be considered for subacromial corticosteroid injections which have been shown to provide significant short-term relief [116,118]. Adjunctive treatment modalities also include acupuncture, electrical stimulation, phonophoresis, iontophoresis, laser therapy and elastic therapeutic taping. Extracorporeal shockwave therapy and barbotage have shown to be beneficial for calcific tendinitis [113]. In cases of refractory symptoms, referral for surgery can be considered however surgical decompression (which is the intervention performed most often) has been shown to have little to no benefit for long-term pain [113,119,120].

#### 3.2.3. Glenohumeral Joint Adhesive Capsulitis (Frozen Shoulder)

Pathophysiology: More commonly referred to as “frozen shoulder”, adhesive capsulitis of the shoulder can be a painful and debilitating condition. Although not completely understood, adhesive capsulitis is thought to develop due to contracture of the glenohumeral joint caused by an inflammatory process contributing to fibrosis [121]. While primary adhesive capsulitis is most often associated with diabetes, treatment for breast cancer may commonly cause secondary adhesive capsulitis [109,122]. One cross sectional study reviewed 785 patients and found an incidence of 3.8% of patients developing adhesive capsulitis following breast cancer surgery [123]. Another study followed 271 women in post-operative months 13–18 and found a cumulative incidence of 10.3% [124]. Aromatase inhibitor therapy, which many breast cancer patients receive to treat or reduce the risk of disease recurrence, may also increase the risk of frozen shoulder.

Diagnosis: Patients will present with shoulder pain and loss of range of motion with external rotation and abduction particularly being affected [125]. Patients should typically have significant loss of range of motion in at least two planes both actively and passively. It may be difficult to distinguish from shoulder impingement syndrome as severe pain may also limit range of motion. A subacromial injection with local anesthetic can help to distinguish the two as pain should be relieved but range of motion still limited when adhesive capsulitis is present [126]. Imaging is typically not required for diagnosis however plain films and MRI may be helpful in ruling out other etiologies of shoulder pain [127,128].

Stages: Frozen shoulder is commonly thought to be a self-limiting disorder running through three stages. The first stage is a painful or freezing stage in which there is progressive stiffness and pain. The second stage is a stiff or frozen stage in which loss of range of motion is at its peak and pain starts to lessen. The third stage is a recovery or thawing stage in which there is gradual return of range of motion. Each stage may last a few to several months with a full course lasting 1–2 years [128].

Treatment: Although frozen shoulder is usually self-limiting, as many patients have persistent deficits, active management may be essential. Pain may initially be controlled with NSAIDs or acetaminophen. Patients may undergo range of motion exercises at home and in some cases skilled physical therapy may be indicated; the evidence for this is mixed and dedicated physical therapy is likely more helpful in the later stages of frozen shoulder than the acute phase [129]. Additionally, there should be a low threshold for early intra-articular corticosteroid injections which have been shown to provide significant short-term relief and long-term improvement in ROM [129,130,131,132]. Other promising treatments may include calcitonin, extracorporeal shockwave therapy, low level laser therapy, hydrodistention and hyaluronic acid injections [133,134]. Surgical referral for manipulation under anesthesia or capsular release can be considered in refractory cases [133].

#### 3.2.4. Myofascial Pain

Pathophysiology: Myofascial pain is an evolving concept that most often describes pain from muscles and their surrounding fascia characterized by myofascial trigger points. Trigger points are taut bands of muscle which are tender and should reproduce pain in characteristic referral patterns [135]. Myofascial pain remains incompletely understood but is thought to arise from a number of factors including muscle overuse, dysfunction at the level of the fascia, peripheral sensitization and central sensitization [136,137]. Pectoral tightness contributes to a protracted and depressed scapula straining muscles and fascia in the neck and upper back leading to myofascial pain [109]. Scapular dyskinesia may also occur secondary to injury of the pectoralis, thoracodorsal, and long thoracic nerves. Furthermore, damaged intercostal nerves may contribute to myofascial pain of the intercostal muscles. One study demonstrated that breast cancer patients who had undergone either mastectomy or lumpectomy had an average of 4.6 trigger points opposed to an average of 1.1 trigger points in breast cancer patients who had not undergone surgery [138]. Another study followed breast cancer patients for 12 months following surgery and found that 44.8% of patients and developed myofascial pain syndrome [139].

Diagnosis: Myofascial pain is most often characterized by the presence of trigger points. There are no standardized criteria for the diagnosis of a trigger point although the presence of a taut band of muscle that is tender is enough to begin treatment [140].

Treatment: There are numerous treatments commonly used for myofascial pain. Pharmacologic therapies may include NSAIDs, muscle relaxants, benzodiazepines, serotonin and norepinephrine reuptake inhibitors, tricyclic antidepressants and various topicals including menthol, cannabidiol and lidocaine based creams [141]. The pillars of non-pharmacologic management are education on behavior/activity modification and exercise with a focus on relieving strained muscles through strengthening and stretching of antagonists. Adjunctive therapies may include electrical stimulation, massage, acupuncture, stress reduction and trigger point injections [141]. Dry needling has been shown to reduce pain from chronic trigger points, with the effects lasting beyond six weeks [142,143]. Additionally, there are case reports of botulinum toxin being used for various manifestations of musculoskeletal pain and dystonia in the setting of radiation fibrosis syndrome however further research is needed [144].

#### 3.2.5. Lymphedema

Pathophysiology: Lymphedema occurs due to impairment of the lymphatic system and manifests primarily as a sensation of limb fullness and swelling. In the breast cancer population this lymphatic disruption occurs most commonly from radiation damage and lymphadenectomy [145]. The highest risk of lymphedema is for patients who undergo axillary lymph node dissection with a reported incidence of 19.9% vs. 5.6% for those undergoing sentinel lymph node biopsy [146]. Radiation to regional nodes may further increase risk [146]. While lymphedema is not typically associated with acute pain (pain with swelling may be a harbinger of venous thrombosis or cellulitis), it is frequently accompanied by a sensation of heaviness and discomfort [147].

Diagnosis: Diagnosis is most often made clinically based on a patient’s risk status and a visual assessment of disproportionately sized arms [145]. Imaging is not routinely obtained however if indicated, lymphoscintigraphy is considered the standard modality [147]. Additionally, magnetic resonance lymphography and near-infrared fluorescence imaging with indocyanine green are increasingly being used primarily in research and surgical settings respectively [147].

Treatment: Initial treatment is conservative consisting of complete decongestive therapy (CDT). CDT consists of a reduction phase and a maintenance phase consisting of components including compression wrapping, compression garments, skin care, exercise, education and manual lymphatic drainage [148]. Pneumatic compression devices may be used in conjunction with CDT and may provide a synergistic effect when combined with manual lymphatic drainage [147]. There is increasing evidence for the role of surgical procedures including lymphovenous bypass, lymph node transplants, and debulking procedures which may be considered [149].

### 3.3. Nociplastic Pain

In 2017, the International Association for the Study of Pain (IASP) recognized a third mechanism of pain termed nociplastic pain. Nociplastic pain, which is more commonly referred to as centralized pain, is defined by the IASP as “Pain that arises from altered nociception despite no clear evidence of actual or threatened tissue damage causing the activation of peripheral nociceptors or evidence for disease or lesion of the somatosensory system causing the pain” [150]. Nociplastic pain is thought to be the primary pain mechanism occurring in chronic pain conditions such as fibromyalgia, irritable bowel syndrome and temporomandibular joint disorder [151] and has recently been shown to play a significant role in the breast cancer population as one study demonstrated decreased pain pressure thresholds in women who had undergone lumpectomy or mastectomy [152]. Another study found the predominant pain type in breast cancer patients to be nociplastic pain in 15.4% of cases and to be a component of pain in 44% of cases [153]. There is no standardized method of assessing for the presence of nociplastic pain although the Central Sensitization Inventory and the Fibromyalgia Survey Questionnaire have been used in research settings [153,154]. As research continues to emerge in this area, nociplastic pain will be important to consider in the breast cancer population as treatment strategies differ for nociplastic pain syndromes and often require a multidisciplinary approach [151].

## 4. Integrative Approaches to Chronic Pain after Breast Cancer Treatment

A rapidly growing evidence base of integrative approaches during and after breast cancer treatment has significantly broadened the menu of options available for clinicians caring for these patients. Integrative oncology aims to coordinate the delivery of evidence-based complementary therapies with conventional care [155]. Complementary therapies encompass a broad range of mind and body practices along with lifestyle interventions and are commonly employed by breast cancer survivors [156]. Outcomes are typically best when integrative therapies are tailored to the needs of the individual and when there is an evidence base for the integrative approach for the source of pain in question. For example, increasing evidence has suggested that psychosocial factors, such as catastrophizing, somatization, anxiety, and sleep disturbance, may increase the risk for persistent post-mastectomy pain [31,36]. When considering these factors, endorsing a trial of an intervention that may mitigate these symptoms may be valuable. Mindfulness-based stress reduction is a type of meditation practice based on a structured curriculum that teaches nonjudgmental awareness and a conscious awareness for the current moment. A recent Cochrane Database Review suggested that a mindfulness-based stress reduction program may reduce symptoms of anxiety, depression, and insomnia in the short-term [157]. Another intervention known as mindfulness-based cognitive therapy may have significant, robust, and durable effects on pain intensity based on clinical trials [158]. However, the exact source of pain was not delineated in this study, which may limit how it can be tailored for breast cancer survivors.

A systematic review of randomized clinical trials done among breast cancer patients found that hypnosis may improve pain from a variety of sources, including surgery and radiotherapy [159]. It is unclear how patient selection may play a role in studies involving hypnosis as highly hypnotizable patients report greater benefits from hypnosis [160]. Acupuncture, which is a modality derived from Traditional Chinese Medicine, is believed to increase the production of endogenous analgesic neurotransmitters and modulate the perception of pain [161]. Acupuncture has an increasingly strong evidence base in the management of chronic conditions in cancer survivors, including chemotherapy-induced nausea and vomiting, anxiety, and post-treatment fatigue [162]. While acupuncture has increasing evidence for some sources of pain that may be implicated for the breast cancer survivor, such as with myofascial pain, more research is needed [163]. Music therapy is the use of music as a therapeutic intervention and has been employed by a number of controlled studies for pain in the cancer population in general. While more research is needed, music therapy has been shown to reduce pain with a large effect size with some evidence specifically among women experiencing pain from breast surgery [163].

In order to summarize the available evidence for clinicians and to provide evidence-based guidance on the use of integrative therapies during and after breast cancer treatment, the Society for Integrative Oncology produced an updated clinical practice guideline that was endorsed by the American Society of Clinical Oncology in 2018 [162]. Based on this comprehensive review, acupuncture, healing touch, hypnosis, and music therapy “can be considered for the management of pain”, which was felt to have Grade C evidence (“Recommends selectively offering or providing this service to individual patients based on professional judgment and patient preferences”) [162]. For the most part, all of these interventions can be considered exceedingly safe, although clinicians are obligated to consider cost and time utilization concerns when making recommendations.

## 5. Conclusions

There are myriad causes of chronic pain following treatment of breast cancer that may be difficult to distinguish and which may occur concurrently with one another. Oftentimes these causes are grouped together and uniformly termed post-mastectomy pain syndrome, which may be problematic as the underlying issue is not addressed. By rooting out the specific source of pain, which can usually be done by taking a thorough history and physical exam, targeted treatments can be applied.

## Figures and Tables

**Table 1:** Recommendations for the pharmacologic management of neuropathic pain from the Canadian Pain Society (CPS), the Western Australian Therapeutic Advisory Group (WATAG), the Japan Society of Pain Clinicians (JSPC) and the International Association for the Study of Pain (NeuPSIG).

	CPS [38]	WATAG [39]	JSPC [40]	NeuPSIG [41]
1st Line	Gabapentinoids, TCA’s, SNRI’s	TCA’s, SNRI’s	Pregabalin/gabapentin, TCA’s, SNRI’s	SNRI’s, TCA’s, Pregabalin, Gabapentin, Gabapentin Enacarbil
2nd Line	Tramadol, Opioid Analgesics	Pregabalin/Gabapentin	Extract from inflamed cutaneous tissue of rabbits inoculated with vaccinia virus, Tramadol	Tramadol, Capsaicin 8% patches, lidocaine patches
3rd Line	Cannabinoids	Tramadol, Tapentadol	Opioids	Strong opioids, BTX-A
4th Line	SSRI’s, Other anticonvulsants, methadone, topical lidocaine	Opioids		
5th Line		Sodium valproate, Sublingual ketamine		

Tricyclic antidepressant (TCA), serotonin and norepinephrine reuptake inhibitor (SNRI), selective serotonin reuptake inhibitor (SSRI), botulinum toxin A (BTX-A). CYP2D6 inhibitors including duloxetine are contraindicated for patients on tamoxifen as they may decrease serum concentration of active metabolites of tamoxifen.

**Table 2:** Clinical guidelines on neuropathic pain in adults for pharmacological management in non-specialist settings from the National Institute for Health and Care Excellence (NICE).

NICE [42]
First line of amitriptyline, duloxetine, gabapentin or pregabalin Offer one of the remaining above medications if the initial treatment is ineffective Consider tramadol only if acute rescue therapy is needed Consider capsaicin cream for localized pain

## References

[1] Cancer Fact Sheets: 39 All Cancers Fact Sheet. https://gco.iarc.fr/today/data/factsheets/cancers/39-All-cancers-fact-sheet.pdf.

[2] Waks A.G., Winer E.P. (2019). Breast Cancer Treatment: A Review. JAMA.

[3] American Cancer Society (2020). Breast Cancer Facts & Figures 2019–2020.

[4] How Common Is Breast Cancer? Breast Cancer Statistics. https://www.cancer.org/cancer/breast-cancer/about/how-common-is-breast-cancer.html.

[5] Treede R.-D., Rief W., Barke A., Aziz Q., Bennett M.I., Benoliel R., Cohen M., Evers S., Finnerup N.B., First M.B. (2019). Chronic Pain as a Symptom or a Disease: The IASP Classification of Chronic Pain for the International Classification of Diseases (ICD-11). Pain.

[6] Waltho D., Rockwell G. (2016). Post-Breast Surgery Pain Syndrome: Establishing a Consensus for the Definition of Post-Mastectomy Pain Syndrome to Provide a Standardized Clinical and Research Approach—A Review of the Literature and Discussion. Can. J. Surg..

[7] Wood K.M. (1978). Intercostobrachial Nerve Entrapment Syndrome. South. Med. J..

[8] Visnjevac O., Matson B. (2013). Postmastectomy Pain Syndrome: An Unrecognized Annual Billion Dollar National Financial Burden. J. Pain.

[9] Rietman J.S., Dijkstra P.U., Hoekstra H.J., Eisma W.H., Szabo B.G., Groothoff J.W., Geertzen J.H.B. (2003). Late Morbidity after Treatment of Breast Cancer in Relation to Daily Activities and Quality of Life: A Systematic Review. Eur. J. Surg. Oncol..

[10] Sugden E.M., Rezvani M., Harrison J.M., Hughes L.K. (1998). Shoulder Movement after the Treatment of Early Stage Breast Cancer. Clin. Oncol..

[11] Segerström K., Bjerle P., Nyström Å. (1991). Importance of Time in Assessing Arm and Hand Function After Treatment of Breast Cancer. Scand. J. Plast. Reconstr. Surg. Hand Surg..

[12] Kaya T., Karatepe A.G., Günaydn R., Yetiş H., Uslu A. (2010). Disability and Health-Related Quality of Life after Breast Cancer Surgery: Relation to Impairments. South. Med. J..

[13] Swedborg I., Borg G., Sarnelid M. (1981). Somatic Sensation and Discomfort in the Arm of Post-Mastectomy Patients. Scand. J. Rehabil. Med..

[14] Tasmuth T., von Smitten K., Kalso E. (1996). Pain and Other Symptoms during the First Year after Radical and Conservative Surgery for Breast Cancer. Br. J. Cancer.

[15] Hack T.F., Cohen L., Katz J., Robson L.S., Goss P. (1999). Physical and Psychological Morbidity after Axillary Lymph Node Dissection for Breast Cancer. J. Clin. Oncol..

[16] Stevens P.E., Dibble S.L., Miaskowski C. (1995). Prevalence, Characteristics, and Impact of Postmastectomy Pain Syndrome: An Investigation of Women’s Experiences. Pain.

[17] Caffo O., Amichetti M., Ferro A., Lucenti A., Valduga F., Galligioni E. (2003). Pain and Quality of Life after Surgery for Breast Cancer. Breast Cancer Res. Treat..

[18] Beyaz S.G., Ergönenç J.S., Ergönenç T., Sönmez Ö.U., Erkorkmaz Ü., Altintoprak F. (2016). Postmastectomy Pain: A Cross-Sectional Study of Prevalence, Pain Characteristics, and Effects on Quality of Life. Chin. Med. J..

[19] Lee J.S.-J., Hu H.M., Edelman A.L., Brummett C.M., Englesbe M.J., Waljee J.F., Smerage J.B., Griggs J.J., Nathan H., Jeruss J.S. (2017). New Persistent Opioid Use Among Patients with Cancer After Curative-Intent Surgery. JCO.

[20] (2021). United Nations Office on Drugs and Crime Opioids. World Drug Report 2021: Drug Market Trends: Opioids, Cannabis.

[21] The Opioid Epidemic Within the COVID-19 Pandemic: Drug Testing in 2020. https://pubmed.ncbi.nlm.nih.gov/33031013/.

[22] Appa A., Rodda L.N., Cawley C., Zevin B., Coffin P.O., Gandhi M., Imbert E. (2021). Drug Overdose Deaths Before and After Shelter-in-Place Orders During the COVID-19 Pandemic in San Francisco. JAMA Netw. Open.

[23] American Medical Association Issue Brief: Drug Overdose Epidemic Worsened during COVID Pandemic. https://www.ama-assn.org/system/files/2020-12/issue-brief-increases-in-opioid-related-overdose.pdf.

[24] A Crisis on Top of a Crisis: COVID-19 and the Opioid Epidemic. https://www.hsph.harvard.edu/news/features/a-crisis-on-top-of-a-crisis-COVID-19-and-the-opioid-epidemic/.

[25] Granek I., Ashikari R., Foley K. (1984). The Post-Mastectomy Pain Syndrome: Clinical and Anatomical Correlates. Proc. Am. Soc. Clin. Oncol..

[26] Merskey H., Bogduk N., International Association for the Study of Pain (1994). Classification of Chronic Pain: Descriptions of Chronic Pain Syndromes and Definitions of Pain Terms.

[27] Vecht C.J., Van de Brand H.J., Wajer O.J. (1989). Post-Axillary Dissection Pain in Breast Cancer Due to a Lesion of the Intercostobrachial Nerve. Pain.

[28] Jung B.F., Ahrendt G.M., Oaklander A.L., Dworkin R.H. (2003). Neuropathic Pain Following Breast Cancer Surgery: Proposed Classification and Research Update. Pain.

[29] Vilholm O.J., Cold S., Rasmussen L., Sindrup S.H. (2008). The Postmastectomy Pain Syndrome: An Epidemiological Study on the Prevalence of Chronic Pain after Surgery for Breast Cancer. Br. J. Cancer.

[30] Andersen K.G., Kehlet H. (2011). Persistent Pain after Breast Cancer Treatment: A Critical Review of Risk Factors and Strategies for Prevention. J. Pain.

[31] Belfer I., Schreiber K.L., Shaffer J.R., Shnol H., Blaney K., Morando A., Englert D., Greco C., Brufsky A., Ahrendt G. (2013). Persistent Postmastectomy Pain in Breast Cancer Survivors: Analysis of Clinical, Demographic, and Psychosocial Factors. J. Pain.

[32] Kokosis G., Chopra K., Darrach H., Dellon A.L., Williams E.H. (2019). Re-Visiting Post-Breast Surgery Pain Syndrome: Risk Factors, Peripheral Nerve Associations and Clinical Implications. Gland Surg..

[33] Shen J. Clinical Manifestations and Diagnosis of Postmastectomy Pain Syndrome. https://www.uptodate.com/contents/clinical-manifestations-and-diagnosis-of-postmastectomy-pain-syndrome?search=post%20mastectomy%20pain&source=search_result&selectedTitle=2~10&usage_type=default&display_rank=2.

[34] Gärtner R., Jensen M.-B., Nielsen J., Ewertz M., Kroman N., Kehlet H. (2009). Prevalence of and Factors Associated with Persistent Pain Following Breast Cancer Surgery. JAMA.

[35] Mejdahl M.K., Andersen K.G., Gärtner R., Kroman N., Kehlet H. (2013). Persistent Pain and Sensory Disturbances after Treatment for Breast Cancer: Six Year Nationwide Follow-up Study. BMJ.

[36] Tait R.C., Zoberi K., Ferguson M., Levenhagen K., Luebbert R.A., Rowland K., Salsich G.B., Herndon C. (2018). Persistent Post-Mastectomy Pain: Risk Factors and Current Approaches to Treatment. J. Pain.

[37] Chappell A.G., Bai J., Yuksel S., Ellis M.F. (2020). Post-Mastectomy Pain Syndrome: Defining Perioperative Etiologies to Guide New Methods of Prevention for Plastic Surgeons. World J. Plast. Surg..

[38] Moulin D., Boulanger A., Clark A., Clarke H., Dao T., Finley G., Furlan A., Gilron I., Gordon A., Morley-Forster P. (2014). Pharmacological Management of Chronic Neuropathic Pain: Revised Consensus Statement from the Canadian Pain Society. Pain Res. Manag..

[39] Western Australian Therapeutic Advisory Group Guidelines for the Pharmacological Treatment of Neuropathic Pain. https://ww2.health.wa.gov.au/~/media/Files/Corporate/general%20documents/WATAG/Neuropathic-Pain-Guidelines.pdf.

[40] Sumitani M., Sakai T., Matsuda Y., Abe H., Yamaguchi S., Hosokawa T., Fukui S. (2018). Executive Summary of the Clinical Guidelines of Pharmacotherapy for Neuropathic Pain: Second Edition by the Japanese Society of Pain Clinicians. J. Anesth..

[41] Finnerup N.B., Attal N., Haroutounian S., McNicol E., Baron R., Dworkin R.H., Gilron I., Haanpää M., Hansson P., Jensen T.S. (2015). Pharmacotherapy for Neuropathic Pain in Adults: A Systematic Review and Meta-Analysis. Lancet Neurol..

[42] National Institute for Health and Care Excellence (2020). Neuropathic Pain in Adults: Pharmacological Management in Non-Specialist Settings.

[43] Henry B.M., Graves M.J., Pękala J.R., Sanna B., Hsieh W.C., Tubbs R.S., Walocha J.A., Tomaszewski K.A. (2017). Origin, Branching, and Communications of the Intercostobrachial Nerve: A Meta-Analysis with Implications for Mastectomy and Axillary Lymph Node Dissection in Breast Cancer. Cureus.

[44] Vadivelu N., Schreck M., Lopez J., Kodumudi G., Narayan D. (2008). Pain after Mastectomy and Breast Reconstruction. Am. Surg..

[45] Wisotzky E.M., Saini V., Kao C. (2016). Ultrasound-Guided Intercostobrachial Nerve Block for Intercostobrachial Neuralgia in Breast Cancer Patients: A Case Series. PM R.

[46] Wijayasinghe N., Duriaud H.M., Kehlet H., Andersen K.G., Anderson K.G. (2016). Ultrasound Guided Intercostobrachial Nerve Blockade in Patients with Persistent Pain after Breast Cancer Surgery: A Pilot Study. Pain Physician.

[47] Yang A., Nadav D., Legler A., Chen G.H., Hingula L., Puttanniah V., Gulati A. (2021). An Interventional Pain Algorithm for the Treatment of Postmastectomy Pain Syndrome: A Single-Center Retrospective Review. Pain Med..

[48] Neumeister M.W., Winters J.N. (2020). Neuroma. Clin. Plast. Surg..

[49] Wong L. (2001). Intercostal Neuromas: A Treatable Cause of Postoperative Breast Surgery Pain. Ann. Plast. Surg..

[50] Shen J. Postmastectomy Pain Syndrome: Risk Reduction and Management. https://www.uptodate.com/contents/postmastectomy-pain-syndrome-risk-reduction-and-management?search=post-mastectomy%20pain%20syndrome&source=search_result&selectedTitle=1~10&usage_type=default&display_rank=1.

[51] AlSharif S., Ferré R., Omeroglu A., El Khoury M., Mesurolle B. (2016). Imaging Features Associated with Posttraumatic Breast Neuromas. AJR Am. J. Roentgenol..

[52] Causeret A., Lapègue F., Bruneau B., Dreano T., Ropars M., Guillin R. (2019). Painful Traumatic Neuromas in Subcutaneous Fat: Visibility and Morphologic Features with Ultrasound. J. Ultrasound Med..

[53] Tang C., Elder S., Lee D. (2013). A simple intervention to relieve chronic neuropathic post-mastectomy pain. Am. Assoc. Cancer Res..

[54] Helwick C. Postmastectomy Pain Effectively Treated with a Simple Injection. https://ascopost.com/issues/february-15-2014/postmastectomy-pain-effectively-treated-with-a-simple-injection/.

[55] Khoury A.L., Keane H., Varghese F., Hosseini A., Mukhtar R., Eder S.E., Weinstein P.R., Esserman L.J. (2021). Trigger Point Injection for Post-Mastectomy Pain: A Simple Intervention with High Rate of Long-Term Relief. NPJ Breast Cancer.

[56] Juhl A.A., Karlsson P., Damsgaard T.E. (2016). Fat Grafting for Alleviating Persistent Pain after Breast Cancer Treatment: A Randomized Controlled Trial. J. Plast. Reconstr. Aesthet. Surg..

[57] Lisa A.V.E., Murolo M., Maione L., Vinci V., Battistini A., Morenghi E., De Santis G., Klinger M. (2020). Autologous Fat Grafting Efficacy in Treating PostMastectomy Pain Syndrome: A Prospective Multicenter Trial of Two Senonetwork Italia Breast Centers. Breast. J..

[58] Santosa K., Oliver J., Cederna P., Kung T. Regenerative Peripheral Nerve Interfaces for Prevention and Management of Neuromas. https://www.plasticsurgery.theclinics.com/article/S0094-1298(20)30004-3/fulltext.

[59] O’Brien A.L., Kraft C.T., Valerio I.L., Rendon J.L., Spitz J.A., Skoracki R.J. (2020). Targeted Muscle Reinnervation Following Breast Surgery: A Novel Technique. Plast. Reconstr. Surg. Glob. Open.

[60] Hart S.E., Brown D.L. (2021). Dermatosensory Peripheral Nerve Interfaces: Prevention of Pain Recurrence Following Sensory Neurectomy. Hand Clin..

[61] University of Michigan Rogel Cancer Center (2020). Surgical Treatment of Post-Surgical Mastectomy Pain Utilizing the Regenerative Peripheral Nerve Interface (RPNI). https://clinicaltrials.gov/ct2/show/NCT04530526.

[62] Flor H. (2002). Phantom-Limb Pain: Characteristics, Causes, and Treatment. Lancet Neurol..

[63] Markopoulos C.J., Spyropoulou A.C., Zervas I.M., Christodoulou G.N., Papageorgiou C. (2010). Phantom Breast Syndrome: The Effect of in Situ Breast Carcinoma. Psychiatry Res..

[64] Jamison K., Wellisch D.K., Katz R.L., Pasnau R.O. (1979). Phantom Breast Syndrome. Arch. Surg..

[65] Dijkstra P.U., Rietman J.S., Geertzen J.H.B. (2007). Phantom Breast Sensations and Phantom Breast Pain: A 2-Year Prospective Study and a Methodological Analysis of Literature. Eur. J. Pain.

[66] Hsu C., Sliwa J.A. (2004). Phantom Breast Pain as a Source of Functional Loss. Am. J. Phys. Med. Rehabil..

[67] Tytherleigh M.G., Koshy C.E., Evans J. (1998). Phantom Breast Pain. Plast. Reconstr. Surg..

[68] Sanders R.J., Annest S.J. (2014). Thoracic Outlet and Pectoralis Minor Syndromes. Semin. Vasc. Surg..

[69] Ammi M., Péret M., Henni S., Daligault M., Abraham P., Papon X., Enon B., Picquet J. (2018). Frequency of the Pectoralis Minor Compression Syndrome in Patients Treated for Thoracic Outlet Syndrome. Ann. Vasc. Surg..

[70] Harrington S.E., Hoffman J., Katsavelis D. (2020). Measurement of Pectoralis Minor Muscle Length in Women Diagnosed with Breast Cancer: Reliability, Validity, and Clinical Application. Phys. Ther..

[71] Shamley D.R., Srinanaganathan R., Weatherall R., Oskrochi R., Watson M., Ostlere S., Sugden E. (2007). Changes in Shoulder Muscle Size and Activity Following Treatment for Breast Cancer. Breast Cancer Res. Treat..

[72] Goshima K. Overview of Thoracic Outlet Syndromes. https://www.uptodate.com/contents/overview-of-thoracic-outlet-syndromes?search=thoracic%20outlet%20syndrome&source=search_result&selectedTitle=1~56&usage_type=default&display_rank=1#H293627611.

[73] Sanders R.J., Rao N.M. (2010). The Forgotten Pectoralis Minor Syndrome: 100 Operations for Pectoralis Minor Syndrome Alone or Accompanied by Neurogenic Thoracic Outlet Syndrome. Ann. Vasc. Surg..

[74] Borstad J.D., Ludewig P.M. (2006). Comparison of Three Stretches for the Pectoralis Minor Muscle. J. Shoulder Elb. Surg..

[75] Lai C.-C., Chen S.-Y., Yang J.-L., Lin J.-J. (2019). Effectiveness of Stretching Exercise versus Kinesiotaping in Improving Length of the Pectoralis Minor: A Systematic Review and Network Meta-Analysis. Phys. Ther. Sport.

[76] Sanders R.J. (2011). Recurrent Neurogenic Thoracic Outlet Syndrome Stressing the Importance of Pectoralis Minor Syndrome. Vasc. Endovasc. Surg..

[77] Robinson J., Kothari M. Clinical Features and Diagnosis of Cervical Radiculopathy. https://www.uptodate.com/.

[78] Schoenfeld A.J., George A.A., Bader J.O., Caram P.M. (2012). Incidence and Epidemiology of Cervical Radiculopathy in the United States Military: 2000 to 2009. J. Spinal Disord Tech..

[79] Iyer S., Kim H.J. (2016). Cervical Radiculopathy. Curr. Rev. Musculoskelet. Med..

[80] Corey D.L., Comeau D. (2014). Cervical Radiculopathy. Med. Clin. N. Am..

[81] Stubblefield M.D., Custodio C.M. (2006). Upper-Extremity Pain Disorders in Breast Cancer. Arch. Phys. Med. Rehabil..

[82] Thoomes E.J., van Geest S., van der Windt D.A., Falla D., Verhagen A.P., Koes B.W., Thoomes-de Graaf M., Kuijper B., Scholten-Peeters W.G.M., Vleggeert-Lankamp C.L. (2018). Value of Physical Tests in Diagnosing Cervical Radiculopathy: A Systematic Review. Spine J..

[83] Rubinstein S.M., Pool J.J.M., van Tulder M.W., Riphagen I.I., de Vet H.C.W. (2007). A Systematic Review of the Diagnostic Accuracy of Provocative Tests of the Neck for Diagnosing Cervical Radiculopathy. Eur. Spine J..

[84] Woods B.I., Hilibrand A.S. (2015). Cervical Radiculopathy: Epidemiology, Etiology, Diagnosis, and Treatment. J. Spinal Disord. Tech..

[85] Nardin R.A., Patel M.R., Gudas T.F., Rutkove S.B., Raynor E.M. (1999). Electromyography and Magnetic Resonance Imaging in the Evaluation of Radiculopathy. Muscle Nerve.

[86] Dillingham T.R., Annaswamy T.M., Plastaras C.T. (2020). Evaluation of Persons with Suspected Lumbosacral and Cervical Radiculopathy: Electrodiagnostic Assessment and Implications for Treatment and Outcomes (Part I). Muscle Nerve.

[87] Childress M.A., Becker B.A. (2016). Nonoperative Management of Cervical Radiculopathy. Am. Fam. Physician.

[88] Kjaer P., Kongsted A., Hartvigsen J., Isenberg-Jørgensen A., Schiøttz-Christensen B., Søborg B., Krog C., Møller C.M., Halling C.M.B., Lauridsen H.H. (2017). National Clinical Guidelines for Non-Surgical Treatment of Patients with Recent Onset Neck Pain or Cervical Radiculopathy. Eur. Spine J..

[89] Romeo A., Vanti C., Boldrini V., Ruggeri M., Guccione A.A., Pillastrini P., Bertozzi L. (2018). Cervical Radiculopathy: Effectiveness of Adding Traction to Physical Therapy-A Systematic Review and Meta-Analysis of Randomized Controlled Trials. Phys. Ther..

[90] Engel A., King W., MacVicar J. (2014). Standards Division of the International Spine Intervention Society the Effectiveness and Risks of Fluoroscopically Guided Cervical Transforaminal Injections of Steroids: A Systematic Review with Comprehensive Analysis of the Published Data. Pain Med..

[91] Conger A., Cushman D.M., Speckman R.A., Burnham T., Teramoto M., McCormick Z.L. (2020). The Effectiveness of Fluoroscopically Guided Cervical Transforaminal Epidural Steroid Injection for the Treatment of Radicular Pain: A Systematic Review and Meta-Analysis. Pain Med..

[92] House L.M., Barrette K., Mattie R., McCormick Z.L. (2018). Cervical Epidural Steroid Injection: Techniques and Evidence. Phys. Med. Rehabil. Clin. N. Am..

[93] Nikolaidis I., Fouyas I.P., Sandercock P.A., Statham P.F. (2010). Surgery for Cervical Radiculopathy or Myelopathy. Cochrane Database Syst. Rev..

[94] Broekema A.E.H., Groen R.J.M., Simões de Souza N.F., Smidt N., Reneman M.F., Soer R., Kuijlen J.M.A. (2020). Surgical Interventions for Cervical Radiculopathy without Myelopathy: A Systematic Review and Meta-Analysis. J. Bone Joint Surg. Am..

[95] Kuhn J.E., Plancher K.D., Hawkins R.J. (1998). Symptomatic Scapulothoracic Crepitus and Bursitis. J. Am. Acad. Orthop. Surg..

[96] Warth R.J., Spiegl U.J., Millett P.J. (2015). Scapulothoracic Bursitis and Snapping Scapula Syndrome: A Critical Review of Current Evidence. Am. J. Sports Med..

[97] Gaskill T., Millett P.J. (2013). Snapping Scapula Syndrome: Diagnosis and Management. J. Am. Acad. Orthop. Surg..

[98] Boneti C., Arentz C., Klimberg V.S. (2010). Scapulothoracic Bursitis as a Significant Cause of Breast and Chest Wall Pain: Underrecognized and Undertreated. Ann. Surg. Oncol..

[99] Baldawi H., Gouveia K., Gohal C., Almana L., Paul R., Alolabi B., Moro J., Khan M. (2021). Diagnosis and Treatment of Snapping Scapula Syndrome: A Scoping Review. Sports Health.

[100] Kuhne M., Boniquit N., Ghodadra N., Romeo A.A., Provencher M.T. (2009). The Snapping Scapula: Diagnosis and Treatment. Arthroscopy.

[101] Conduah A.H., Baker C.L., Baker C.L. (2010). Clinical Management of Scapulothoracic Bursitis and the Snapping Scapula. Sports Health.

[102] Chang W.H., Im S.H., Ryu J.A., Lee S.C., Kim J.S. (2009). The Effects of Scapulothoracic Bursa Injections in Patients with Scapular Pain: A Pilot Study. Arch. Phys. Med. Rehabil..

[103] Chang W.H., Kim Y.W., Choi S., Lee S.C. (2014). Comparison of the Therapeutic Effects of Intramuscular Subscapularis and Scapulothoracic Bursa Injections in Patients with Scapular Pain: A Randomized Controlled Trial. Rheumatol. Int..

[104] Wang M.L., Miller A.J., Ballard B.L., Botte M.J. (2016). Management of Snapping Scapula Syndrome. Orthopedics.

[105] Son S.A., Lee D.H., Lee Y.O., Lee S.C., Kim K.J., Cho J.Y. (2013). Operative Management in a Patient with Scapulothoracic Bursitis. Korean J. Thorac. Cardiovasc. Surg..

[106] Simons S., Kruse D., Dixon B. Shoulder Impingement Syndrome. https://www.uptodate.com/contents/shoulder-impingement-syndrome?search=shoulder%20impingement%20syndrome&sectionRank=2&usage_type=default&anchor=H13&source=machineLearning&selectedTitle=1~30&display_rank=1#H17.

[107] Brown D., Freeman E., Cuccurullo S., Ng U., Maitin I. (2015). Musculoskeletal Medicine. Physical Medicine and Rehabilitation Board Review.

[108] Harrison A.K., Flatow E.L. (2011). Subacromial Impingement Syndrome. J. Am. Acad. Orthop. Surg..

[109] Cheville A.L., Tchou J. (2007). Barriers to Rehabilitation Following Surgery for Primary Breast Cancer. J. Surg. Oncol..

[110] Ebaugh D., Spinelli B., Schmitz K.H. (2011). Shoulder Impairments and Their Association with Symptomatic Rotator Cuff Disease in Breast Cancer Survivors. Med. Hypotheses.

[111] Lang A.E., Dickerson C.R., Kim S.Y., Stobart J., Milosavljevic S. (2019). Impingement Pain Affects Kinematics of Breast Cancer Survivors in Work-Related Functional Tasks. Clin. Biomech..

[112] Greenberg D.L. (2014). Evaluation and Treatment of Shoulder Pain. Med. Clin. N. Am..

[113] Diercks R., Bron C., Dorrestijn O., Meskers C., Naber R., de Ruiter T., Willems J., Winters J., van der Woude H.J. (2014). Dutch Orthopaedic Association Guideline for Diagnosis and Treatment of Subacromial Pain Syndrome: A Multidisciplinary Review by the Dutch Orthopaedic Association. Acta Orthop..

[114] Gismervik S.Ø., Drogset J.O., Granviken F., Rø M., Leivseth G. (2017). Physical Examination Tests of the Shoulder: A Systematic Review and Meta-Analysis of Diagnostic Test Performance. BMC Musculoskelet. Disord.

[115] Pesquer L., Borghol S., Meyer P., Ropars M., Dallaudière B., Abadie P. (2018). Multimodality Imaging of Subacromial Impingement Syndrome. Skelet. Radiol..

[116] Steuri R., Sattelmayer M., Elsig S., Kolly C., Tal A., Taeymans J., Hilfiker R. (2017). Effectiveness of Conservative Interventions Including Exercise, Manual Therapy and Medical Management in Adults with Shoulder Impingement: A Systematic Review and Meta-Analysis of RCTs. Br. J. Sports. Med..

[117] Ravichandran H., Janakiraman B., Gelaw A.Y., Fisseha B., Sundaram S., Sharma H.R. (2020). Effect of Scapular Stabilization Exercise Program in Patients with Subacromial Impingement Syndrome: A Systematic Review. J. Exerc. Rehabil..

[118] Penning L.I.F., de Bie R.A., Walenkamp G.H.I.M. (2012). The Effectiveness of Injections of Hyaluronic Acid or Corticosteroid in Patients with Subacromial Impingement: A Three-Arm Randomised Controlled Trial. J. Bone Joint Surg. Br..

[119] Khan M., Alolabi B., Horner N., Bedi A., Ayeni O.R., Bhandari M. (2019). Surgery for Shoulder Impingement: A Systematic Review and Meta-Analysis of Controlled Clinical Trials. Can. Med Assoc. Open Access J..

[120] Paavola M., Kanto K., Ranstam J., Malmivaara A., Inkinen J., Kalske J., Savolainen V., Sinisaari I., Taimela S., Järvinen T.L. (2021). Subacromial Decompression versus Diagnostic Arthroscopy for Shoulder Impingement: A 5-Year Follow-up of a Randomised, Placebo Surgery Controlled Clinical Trial. Br. J. Sports Med..

[121] Le H.V., Lee S.J., Nazarian A., Rodriguez E.K. (2017). Adhesive Capsulitis of the Shoulder: Review of Pathophysiology and Current Clinical Treatments. Shoulder Elb..

[122] Zreik N.H., Malik R.A., Charalambous C.P. (2016). Adhesive Capsulitis of the Shoulder and Diabetes: A Meta-Analysis of Prevalence. Muscles Ligaments Tendons J..

[123] Cho C.-H., Lee K.-L., Cho J., Kim D. (2020). The Incidence and Risk Factors of Frozen Shoulder in Patients with Breast Cancer Surgery. Breast J..

[124] Yang S., Park D.H., Ahn S.H., Kim J., Lee J.W., Han J.Y., Kim D.K., Jeon J.Y., Choi K.H., Kim W. (2017). Prevalence and Risk Factors of Adhesive Capsulitis of the Shoulder after Breast Cancer Treatment. Support Care Cancer.

[125] Fields B.K.K., Skalski M.R., Patel D.B., White E.A., Tomasian A., Gross J.S., Matcuk G.R. (2019). Adhesive Capsulitis: Review of Imaging Findings, Pathophysiology, Clinical Presentation, and Treatment Options. Skelet. Radiol..

[126] Prestgaard T. Frozen Shoulder (Adhesive Capsulitis). https://www.uptodate.com/contents/frozen-shoulder-adhesive-capsulitis?search=adhesive%20capsulitis&source=search_result&selectedTitle=1~39&usage_type=default&display_rank=1#H1568975.

[127] Neviaser A.S., Neviaser R.J. (2011). Adhesive Capsulitis of the Shoulder. J. Am. Acad. Orthop. Surg..

[128] Ramirez J. (2019). Adhesive Capsulitis: Diagnosis and Management. Am. Fam. Physician.

[129] Page M.J., Green S., Kramer S., Johnston R.V., McBain B., Chau M., Buchbinder R. (2014). Manual Therapy and Exercise for Adhesive Capsulitis (Frozen Shoulder). Cochrane Database Syst. Rev..

[130] Challoumas D., Biddle M., McLean M., Millar N.L. (2020). Comparison of Treatments for Frozen Shoulder: A Systematic Review and Meta-Analysis. JAMA Netw. Open.

[131] Song A., Higgins L.D., Newman J., Jain N.B. (2014). Glenohumeral Corticosteroid Injections in Adhesive Capsulitis: A Systematic Search and Review. PM R.

[132] Wang W., Shi M., Zhou C., Shi Z., Cai X., Lin T., Yan S. (2017). Effectiveness of Corticosteroid Injections in Adhesive Capsulitis of Shoulder: A Meta-Analysis. Medicine.

[133] Redler L.H., Dennis E.R. (2019). Treatment of Adhesive Capsulitis of the Shoulder. J. Am. Acad. Orthop. Surg..

[134] Page M.J., Green S., Kramer S., Johnston R.V., McBain B., Buchbinder R. (2014). Electrotherapy Modalities for Adhesive Capsulitis (Frozen Shoulder). Cochrane Database Syst. Rev..

[135] Travell J., Simons D. (1996). Travell & Simons’ Trigger Point Flip Charts.

[136] Stecco A., Gesi M., Stecco C., Stern R. (2013). Fascial Components of the Myofascial Pain Syndrome. Curr. Pain Headache Rep..

[137] Shah J.P., Thaker N., Heimur J., Aredo J.V., Sikdar S., Gerber L. (2015). Myofascial Trigger Points Then and Now: A Historical and Scientific Perspective. PM R.

[138] Fernández-Lao C., Cantarero-Villanueva I., Fernández-de-Las-Peñas C., Del-Moral-Ávila R., Menjón-Beltrán S., Arroyo-Morales M. (2012). Development of Active Myofascial Trigger Points in Neck and Shoulder Musculature Is Similar after Lumpectomy or Mastectomy Surgery for Breast Cancer. J. Bodyw. Mov. Ther..

[139] Torres Lacomba M., Mayoral del Moral O., Coperias Zazo J.L., Gerwin R.D., Goñí A.Z. (2010). Incidence of Myofascial Pain Syndrome in Breast Cancer Surgery: A Prospective Study. Clin. J. Pain.

[140] Gerwin R.D. (2014). Diagnosis of Myofascial Pain Syndrome. Phys. Med. Rehabil. Clin. N. Am..

[141] Borg-Stein J., Iaccarino M.A. (2014). Myofascial Pain Syndrome Treatments. Phys. Med. Rehabil. Clin. N. Am..

[142] Gerber L.H., Shah J., Rosenberger W., Armstrong K., Turo D., Otto P., Heimur J., Thaker N., Sikdar S. (2015). Dry Needling Alters Trigger Points in the Upper Trapezius Muscle and Reduces Pain in Subjects with Chronic Myofascial Pain. PM R.

[143] Gerber L.H., Sikdar S., Aredo J.V., Armstrong K., Rosenberger W.F., Shao H., Shah J.P. (2017). Beneficial Effects of Dry Needling for Treatment of Chronic Myofascial Pain Persist for 6 Weeks After Treatment Completion. PM R.

[144] Stubblefield M.D., Levine A., Custodio C.M., Fitzpatrick T. (2008). The Role of Botulinum Toxin Type A in the Radiation Fibrosis Syndrome: A Preliminary Report. Arch. Phys. Med. Rehabil..

[145] Rockson S.G. (2018). Lymphedema after Breast Cancer Treatment. N. Engl. J. Med..

[146] Gillespie T.C., Sayegh H.E., Brunelle C.L., Daniell K.M., Taghian A.G. (2018). Breast Cancer-Related Lymphedema: Risk Factors, Precautionary Measures, and Treatments. Gland Surg..

[147] Hutchinson N. (2018). Evaluation and Management of Edema and Lymphedema in the Cancer Patient. Cancer Rehabilitation.

[148] McLaughlin S.A., Brunelle C.L., Taghian A. (2020). Breast Cancer-Related Lymphedema: Risk Factors, Screening, Management, and the Impact of Locoregional Treatment. J. Clin. Oncol..

[149] Schaverien M.V., Coroneos C.J. (2019). Surgical Treatment of Lymphedema. Plast. Reconstr. Surg..

[150] International Association for the Study of Pain (1994). Terminology.

[151] Clauw D.J. (2015). Fibromyalgia and Related Conditions. Mayo Clin. Proc..

[152] Fernández-Lao C., Cantarero-Villanueva I., Fernández-de-las-Peñas C., Del-Moral-Ávila R., Menjón-Beltrán S., Arroyo-Morales M. (2011). Widespread Mechanical Pain Hypersensitivity as a Sign of Central Sensitization after Breast Cancer Surgery: Comparison between Mastectomy and Lumpectomy. Pain Med..

[153] Leysen L., Adriaenssens N., Nijs J., Pas R., Bilterys T., Vermeir S., Lahousse A., Beckwée D. (2019). Chronic Pain in Breast Cancer Survivors: Nociceptive, Neuropathic, or Central Sensitization Pain?. Pain Pract..

[154] Brummett C.M., Urquhart A.G., Hassett A.L., Tsodikov A., Hallstrom B.R., Wood N.I., Williams D.A., Clauw D.J. (2015). Characteristics of Fibromyalgia Independently Predict Poorer Long-Term Analgesic Outcomes Following Total Knee and Hip Arthroplasty. Arthritis Rheumatol..

[155] Witt C.M., Balneaves L.G., Cardoso M.J., Cohen L., Greenlee H., Johnstone P., Kücük Ö., Mailman J., Mao J.J. (2017). A Comprehensive Definition for Integrative Oncology. J. Natl. Cancer Inst. Monogr..

[156] Greenlee H., Kwan M.L., Ergas I.J., Sherman K.J., Krathwohl S.E., Bonnell C., Lee M.M., Kushi L.H. (2009). Complementary and Alternative Therapy Use before and after Breast Cancer Diagnosis: The Pathways Study. Breast Cancer Res. Treat..

[157] Schell L.K., Monsef I., Wöckel A., Skoetz N. (2019). Mindfulness-Based Stress Reduction for Women Diagnosed with Breast Cancer. Cochrane Database Syst. Rev..

[158] Johannsen M., O’Connor M., O’Toole M.S., Jensen A.B., Højris I., Zachariae R. (2016). Efficacy of Mindfulness-Based Cognitive Therapy on Late Post-Treatment Pain in Women Treated for Primary Breast Cancer: A Randomized Controlled Trial. J. Clin. Oncol..

[159] Cramer H., Lauche R., Paul A., Langhorst J., Kümmel S., Dobos G.J. (2015). Hypnosis in Breast Cancer Care: A Systematic Review of Randomized Controlled Trials. Integr. Cancer Ther..

[160] Butler L.D., Koopman C., Neri E., Giese-Davis J., Palesh O., Thorne-Yocam K.A., Dimiceli S., Chen X.-H., Fobair P., Kraemer H.C. (2009). Effects of Supportive-Expressive Group Therapy on Pain in Women with Metastatic Breast Cancer. Health Psychol..

[161] Deng G. (2019). Integrative Medicine Therapies for Pain Management in Cancer Patients. Cancer J..

[162] Lyman G.H., Greenlee H., Bohlke K., Bao T., DeMichele A.M., Deng G.E., Fouladbakhsh J.M., Gil B., Hershman D.L., Mansfield S. (2018). Integrative Therapies During and After Breast Cancer Treatment: ASCO Endorsement of the SIO Clinical Practice Guideline. J. Clin. Oncol..

[163] Tola Y.O., Chow K.M., Liang W. (2021). Effects of Non-Pharmacological Interventions on Preoperative Anxiety and Postoperative Pain in Patients Undergoing Breast Cancer Surgery: A Systematic Review. J. Clin. Nurs..

